# Amlodipine alleviates renal ischemia/reperfusion injury in rats through Nrf2/Sestrin2/PGC-1α/TFAM Pathway

**DOI:** 10.1186/s40360-023-00722-6

**Published:** 2023-12-21

**Authors:** Hadi Shirzad, Seyed Amin Mousavinezhad, Mohammad Panji, Moin Ala

**Affiliations:** Research Center for Life, Health Sciences & Biotechnology of the Police, Directorate of Health, Rescue & Treatment, Police Headquarters, Tehran, Iran

**Keywords:** Renal ischemia/reperfusion, Amlodipine, Nrf2, Sestrin2, PGC-1α

## Abstract

**Background:**

Previously, observational studies showed that amlodipine can mitigate calcineurin inhibitor- and contrast-induced acute kidney injury (AKI). Herein, we aimed to measure the effect of amlodipine on renal ischemia/reperfusion (I/R) injury and find the underlying mechanisms.

**Materials and methods:**

Bilateral renal I/R was induced by clamping the hilum of both kidneys for 30 min. The first dose of amlodipine 10 mg/kg was gavaged before anesthesia. The second dose of amlodipine was administered 24 h after the first dose. Forty-eight hours after I/R, rats were anesthetized, and their blood and tissue specimens were collected.

**Results:**

Amlodipine significantly decreased the elevated serum levels of creatinine and blood urea nitrogen (BUN) and mitigated tissue damage in hematoxylin & eosin (H&E) staining. Amlodipine strongly reduced the tissue levels of malondialdehyde (MDA), interleukin 1β (IL1β), and tumor necrosis factor α (TNF-α). Amlodipine enhanced antioxidant defense by upregulating nuclear factor erythroid 2-related factor 2 (Nrf2) and Sestrin2. Furthermore, amlodipine significantly improved mitochondrial biogenesis by promoting Sestrin2/peroxisome proliferator-activated receptor-gamma coactivator (PGC-1α)/mitochondrial transcription factor A (TFAM) pathway. It also enhanced autophagy and attenuated apoptosis, evidenced by increased LC3-II/LC3-I and bcl2/bax ratios after renal I/R.

**Conclusion:**

These findings suggest that amlodipine protects against renal I/R through Nrf2/Sestrin2/PGC-1α/TFAM Pathway.

**Supplementary Information:**

The online version contains supplementary material available at 10.1186/s40360-023-00722-6.

## Introduction

Analysis of large-sized databases showed that there is an increasing trend in the incidence of AKI [1]. Furthermore, AKI is a common finding amongt hospitalized patients, with a prevalence of nearly up to 20% and 33% among hospitalized adults and children with an acute illness, respectively [2]. Renal ischemia and hypoperfusion are prototypical etiologies for AKI [3]. Renal I/R leads to podocyte and tubulointerstitial injury, mesangial proliferation, and glomerulosclerosis, and impairs renal function [3]. Recent studies have shown that AKI is a predictor of chronic kidney disease (CDK) [2]. Because of the high incidence and burden of AKI and its association with CKD, effective management is urgently needed. The current management protocol for AKI is based on prevention, removal of primary etiology, early fluid resuscitation, and renal replacement therapy [4, 5], and the current guideline does not support pharmacological management of AKI [5].

Previously, it has been found that calcium channel blockade may improve renal ischemia; however, results are too preliminary and the underlying molecular mechanisms are poorly understood [6, 7]. Recently, several observational studies indicated the protective effect of amlodipine on human AKI. Interestingly, a multivariate analysis of data from 435 adult patients who underwent primary isolated coronary artery bypass graft surgery showed that no intake of calcium-channel blockers is an independent and strong risk factor for AKI after surgery (95% confidence interval (CI), odds ratio (OR) 4.892 (1.496–16.025)) [8]. A retrospective study of 350 patients who underwent allogeneic hematopoietic stem cell transplantation revealed that amlodipine can ameliorate calcineurin inhibitor-induced nephrotoxicity [9]. Amlodipine-treated patients had a milder decrease in creatinine clearance 30 days (− 17.4 mL/min vs − 33.8 mL/min, *p* < 0.001) and 180 days (− 40.9 vs − 50.6 mL/min, *p* = 0.005) after transplantation [9]. Meanwhile, the incidence of hospitalization with AKI was remarkably lower in patients who received amlodipine, compared with non-users [9]. Yin et al*.* reported that the incidence of contrast-induced nephropathy was significantly (adjusted OR 0.577, *P* = 0.007; matched and propensity‐score OR 0.687, *P* = 0.015) lower among 868 hypertensive patients with amlodipine pretreatment, compared with 1,798 hypertensive patients without amlodipine pretreatment [10]. Moreover, amlodipine pretreatment was associated with markedly longer overall survival (95% CI, HR 0.623 (0.430–0.908)) [10].

Insufficient blood and oxygen supply during ischemia and subsequent mitochondrial dysfunction can lead to oxidative burst and uncontrolled production of reactive oxygen species (ROS) in the reperfusion phase [11]. ROS overproduction can induce cell death by damaging DNA, cell membrane, and protein structure [11]. In addition, upregulating Nrf2, a major transcription factor for many endogenous antioxidants, or PGC-1α, an inducer of mitochondrial biogenesis, was shown to mitigate kidney injury after renal I/R [11–13]. Increased expression of Sestrin2, an endogenous antioxidant and a metabolic regulator, was also found to markedly mitigate kidney damage by oxidative stress [14].

Previous studies reported that amlodipine can ameliorate oxidative stress after organ I/R and strengthen the endogenous antioxidant defense; however, the underlying mechanisms and signaling pathways remain unknown [15, 16]. This study aimed to measure the effect of amlodipine on renal I/R injury in rats and to uncover the underlying molecular mechanisms with a particular focus on the potential role of Sestrin2, Nrf2, and PGC-1α.

## Materials and method

### Animals and grouping

Eighteen healthy male Wistar rats weighing 200–250 g were purchased from the Pharmacology Department of Tehran University of Medical Sciences (TUMS). Rats had free access to standard food and water. They were kept in a temperature-controlled room (25 ± 2 ֯C) with a 12:12 h cycle of light and dark. After acclimatization to their environment, the study began. Rats were treated humanely and sacrificed using a CO_2_ chamber after tissue extraction. Rats were randomly divided into three groups (*n* = 6 per group), including healthy control, ischemic control, and amlodipine-treated group. The healthy control group underwent surgery for blood sampling and tissue biopsy, while the ischemic control group and the amlodipine-treated group underwent I/R injury. Animals were anesthetized with ketamine (87 mg/kg) and xylazine (13 mg/kg) before induction of I/R injury and tissue extraction [17]. Rats were treated according to the Guide for the Care and Use of Laboratory Animals (8th edition, National Academies Press) and institutional guidelines for animal care and use (Department of Pharmacology, School of Medicine, Tehran University of Medical Sciences, Tehran, Iran). This study has been approved by the Ethics Committee of Vice President of Health, Relief, and Treatment of the Police Command of the Islamic Republic of Iran affiliated with Shahid Beheshti University of Medical Sciences (IR.SBMU.TEB.POLICE.ERC.1402.057).

### I/R injury and treatment schedule

Renal I/R injury was induced 48 h before tissue biopsy. To induce I/R injury, rats were anesthetized and their abdomens were shaved. Bilateral flank incisions were used to provide access to both kidneys. The renal hilum was ligated with a bulldog clamp for 30 min. Ischemia was induced in both kidneys and the clamp was reopened after 30 min. The incisions were sutured and the animals remained under close observation until awakening. The total duration of surgery was approximately 40 min, including 30 min of ischemia, 5 min of opening the abdominal wall and exploring renal arteries, and 5 min of suturing the abdominal wall. Rapid and severe pallor and discoloration of the kidney were considered as successful induction of ischemia.

**Fig. 1:**
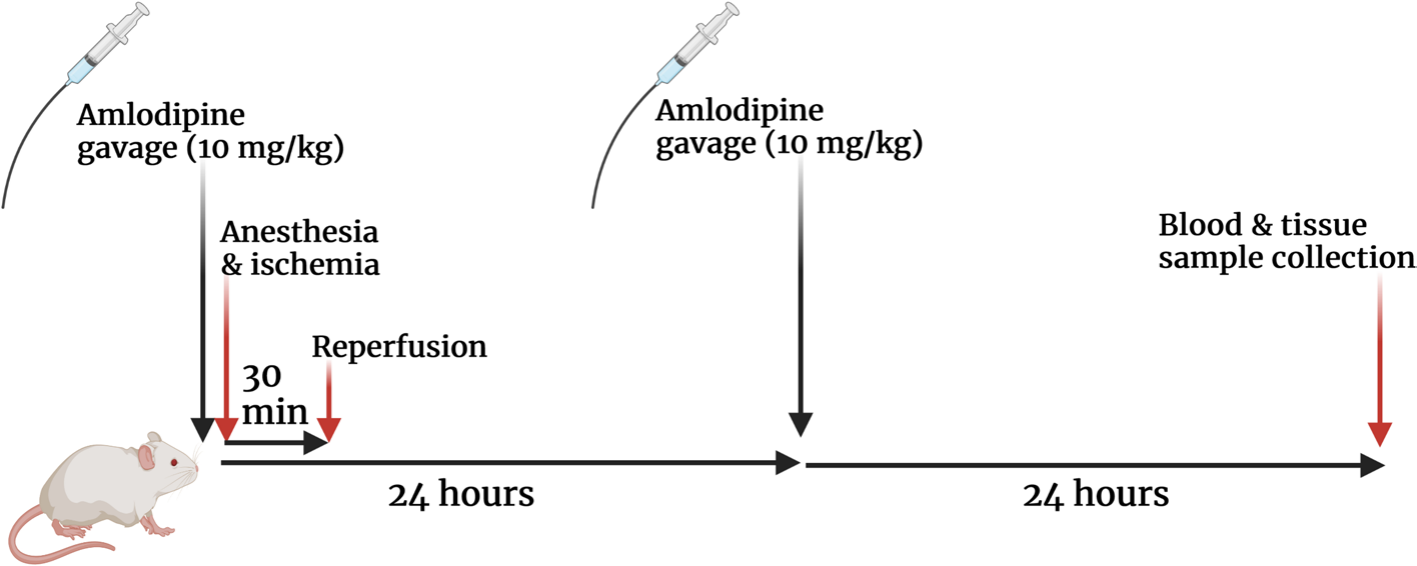
The timeline of animal surgery and treatment schedule

Amlodipine was administered via gavage and normal saline was used as the vehicle. The highest and most effective dose of amlodipine (Alborz Darou, Iran) in previous studies of organ ischemia/reperfusion in rats that had no side effects (10 mg/kg) was administered in this study [16]. The first dose of amlodipine 10 mg/kg was administered 10 min before anesthesia by gavage and the same dose of amlodipine was repeated 24 h after the first dose. The control group received oral gavage of normal saline with the same volume and timing as the treated group. Rats were anesthetized 48 h after I/R injury and after abdominal incision, blood samples were taken from their renal artery (Fig. 1) [18]. In addition, kidneys were harvested for histological and molecular measurements.

### Blood and tissue sample collection

Rats were anesthetized 48 h after I/R injury. After opening the abdominal wall, blood samples were taken from renal arteries and collected in clot-activator tubes. Serum samples were collected after centrifugation (Fig. 1) [18]. In addition, both kidneys were harvested for histological and molecular measurements. After washing in phosphate buffer saline, one kidney was snap-frozen at -80 °C for molecular assay, and another was fixed in formaldehyde 4% for histological assessment.

### Serologic tests and histological measurements

Blood samples were centrifuged (3000 rounds per minute (rpm) for 5 min), frozen, and kept at -20 °C until the serum level of creatinine and BUN were analyzed by a fully automated Hitachi analyzer (*n* = 6). To measure the histological changes, tissue biopsies were fixed in formaldehyde 4%. After tissue processing and slicing, each sample was stained with H&E. An expert pathologist blinded to the samples interpreted them. Based on the EGTI histology scoring system for renal I/R injury [19] (Table 1), each sample received a score from each section, including tubular, glomerular, endothelial, and tubulointerstitial features. A sum of these scores was calculated for each sample. Groups then were compared according to their EGTI histology scores.

**Table 1:** EGTI histology scoring system for renal ischemia/reperfusion injury defined by Chavez et al. ^30^

Tissue type	Damage	Score
**Tubular**	No damage	0
	Loss of brush border (BB) in less than 25% of tubular cells. Integrity of basal membrane	1
	Loss of BB in more than 25% of tubular cells, thickened basal membrane	2
	(Plus) Inflammation, cast formation, necrosis up to 60% of tubular cells	3
	(Plus) Necrosis in more than 60% of tubular cells	4
**Endothelial**	No damage	0
	Endothelial swelling	1
	Endothelial disruption	2
	Endothelial loss	3
**Glomerular**	No damage	0
	Thickening of Bowman capsule	1
	Retraction of glomerular tuft	2
	Glomerular fibrosis	3
**Tubulointerstitial**	No damage	0
	Inflammation, hemorrhage in less than 25% of tissue	1
	Inflammation, hemorrhage in less than 25% of tissue plus necrosis in less than 25% of tissue	2
	Necrosis up to 60%	3
	Necrosis more than 60%	4

### Enzyme-linked immunosorbent assay (ELISA)

Kidney tissue samples were kept at -80 °C until the assay (*n* = 5). After homogenization, the tissue levels of TNF-α and IL1β were determined by using Rat TNF-α DuoSet® ELISA Development kit (catalog number: DY510) and Rat IL-1β/IL-1F2 DuoSet® ELISA Development kit (catalog number: DY501). Kits were used according to the manufacturer’s instructions. After plate preparation, 100 μL of samples or standards were added to the reagent diluent and incubated for 2 h at room temperature. Each well was aspirated and washed with wash buffer. Then, 100 μL of diluted detection antibody was added to each well. The plate was washed again, 100 μL of working dilution of streptavidin-HRP B was added to each well, and the plate was dark incubated at room temperature for 20 min. Thereafter, the plate was washed again, 100 μL of substrate solution was added to each well, and the plate was dark incubated at room temperature for 20 min. In the next step, 50 μL of stop solution was added to each well and the plate was gently tapped to ensure mixing. The optical density of each well was determined by a microplate reader set to 540 nm. The standard curve was drawn and the tissue levels of TNF-α and IL1β were calculated.

### MDA assay

Kidney tissue was used for measuring tissue levels of MDA, as a marker of lipid peroxidation and oxidative stress [20] (*n* = 5). A Biocore (ZellBio) MDA assay kit (catalog number: ZB-MDA96) was used to measure tissue levels of MDA. MDA level measurement was done following manufacturer’s instruction. First of all, 100 μL of standards or samples were added to each test tube. Then, 100 μL of diluted BCD-R4 was added. Thereafter, 200 μL chromogenic solution was added to each test tube. The mixture was heated for one hour in a boiling water bath, cooled in an ice bath, and centrifuged for 10 min at 10,000 rpm. Finally, 200 μL of pink color supernatant was transferred into a microplate and the absorbance was read at 535 nm. The standard curve was drawn and MDA levels were calculated accordingly.

### Western blotting

Kidney tissue samples were snap-frozen and kept at -80 °C until assay. The lysis buffer, including tris–HCL (500µL, PH = 8), EDTA (0.003 g), NaCl (0.08 g), sodium deoxycholate (0.025 g), SDS (0.01 g), protease inhibitor cocktail (1 tablet) and triton (NP40(1%)) (10µL) was mixed with tissue homogenate (*n* = 3). The mixture was centrifuged (Eppendorph 5415 R) at 12,000 rpm and 4 °C for 10 min. The supernatant was extracted and kept at -20 °C. Bradford protein assay using Bradford solution (Coomassie Blue G250 (5 mg), ethanol 95% (2.5 mg), Phosphoric acid (5 mg), distilled water (up to 50 mL)) was utilized to determine the concentration of total protein in each sample. Samples reached the same concentration of total protein before adding them to wells. At the end of the running phase, protein bands were transferred from the SDS page onto the PVDF membrane. Then, PVDF membrane was incubated with primary antibodies (β-actin (SANTA CRUZ, sc-47778, 1:300), bax (SANTA CRUZ, sc-7480, 1:300), bcl2 (SANTA CRUZ, sc-492, 1:300), TFAM (SANTA CRUZ, sc-166965, 1:300), Nrf2 (SANTA CRUZ, sc-365949, 1:300), PGC1-α (Abcam, ab54481, 1:300), Sestrin2 (SANTA CRUZ, sc-101249, 1:300), phosphorylated AMP-activated protein kinase (p-AMPK) (SANTA CRUZ, sc-33524, 1:300), AMPK (SANTA CRUZ, sc-74461, 1:300), and LC3B (Cell Signaling, LC3B Antibody #2775, 1:300) for 16 h. Afterward, the membrane was washed with TBS-T buffer, then incubated with the secondary antibody (SANTA CRUZ, sc-2357, 1:1000) at room temperature for 75 min. An open-source version of Image J software was utilized to quantify the optical density of each protein band.

### Statistical analysis

Data were analyzed by GraphPad Prism version 6.07. One-way analysis of variance (ANOVAs) followed by post hoc Tukey’s tests were used to compare the group. Kruskal–Wallis test and Dunn’s posthoc test were used only for comparing histological scores. Charts are shown as mean ± standard error of the mean (SEM). Differences between groups were considered significant when *p*-value < 0.05.

## Results

### Amlodipine-treated rats had significantly better renal function after I/R injury

**Fig. 2:**
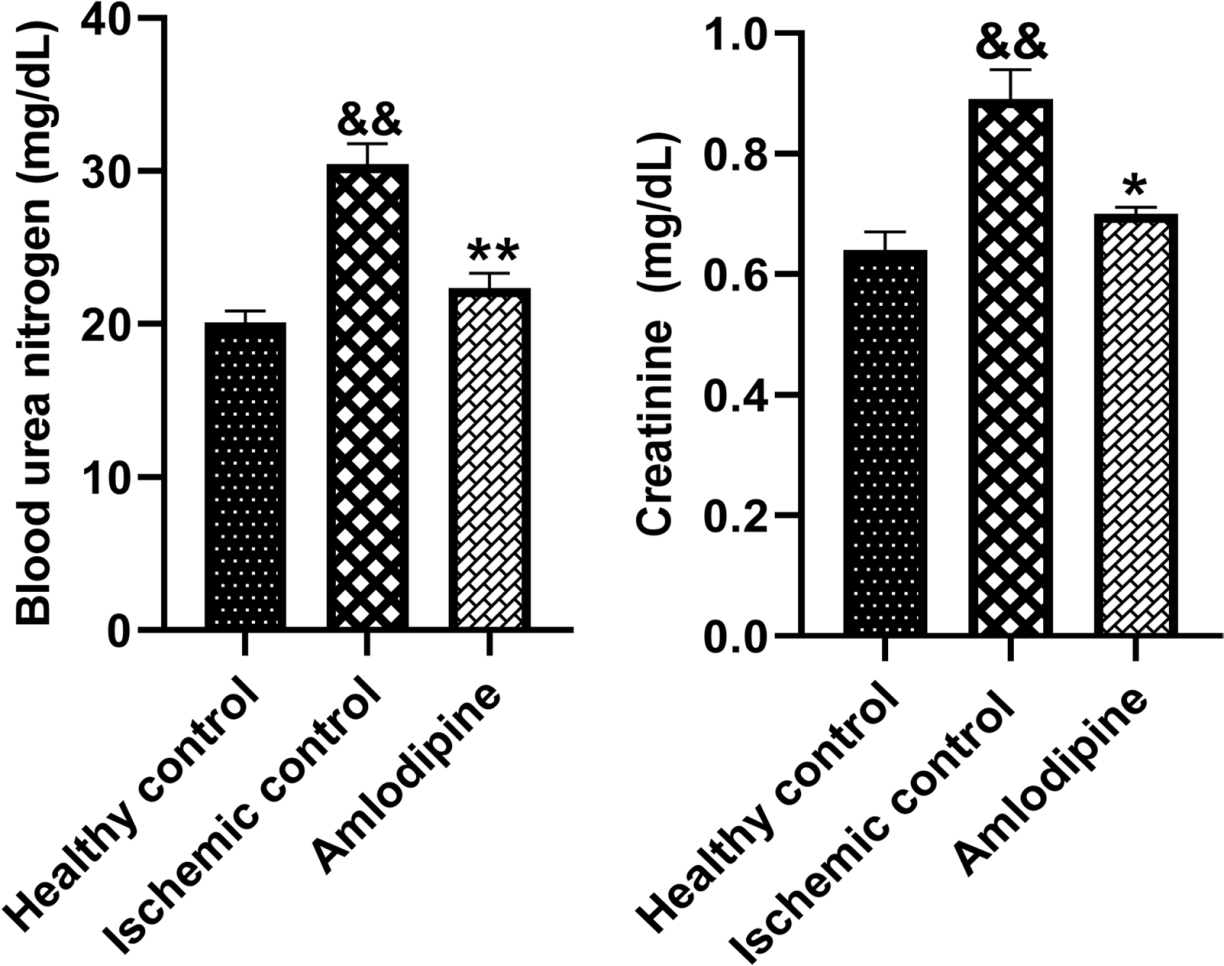
The effect of amlodipine on renal function after renal I/R injury. A significant rise was observed in serum creatinine (&&, *p* < 0.01) and BUN (&&, *p* < 0.01) levels, 48 h after renal I/R injury. Treatment with amlodipine decreased serum levels of creatinine (*, *p* < 0.05) and BUN (**, *p* < 0.01). One-way analysis of variance (ANOVAs) followed by post hoc Tukey’s tests was used to compare the groups. Charts are shown as mean ± standard error of the mean (SEM) (*n* = 6)

Rats were anesthetized and their abdomen was reopened, 48 h after I/R injury. Before nephrectomy, blood samples were collected from renal arteries. A fully automated Hitachi analyzer was used to measure serum creatinine and BUN levels. Renal I/R injury has been associated with a marked increase in serum creatinine (&&, *p* < 0.01) and BUN (&&, *p* < 0.01). The amlodipine-treated group had significantly lower serum levels of creatinine (*, *p* < 0.05) and BUN (**, *p* < 0.01) (Fig. 2).

### Amlodipine significantly ameliorated tissue damage after renal I/R injury

**Fig. 3:**
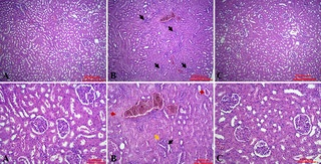
The effect of renal I/R injury and amlodipine on tissue structure. Renal I/R injury severely affected the tissue. Inflammation, hemorrhage, necrosis of tubular cells (red arrow), thickening of Bowman capsule (yellow arrow), retraction of glomerular tuft (black arrow), and glomerular fibrosis were abundantly found in the ischemic control group (**B**), compared with the healthy control group (**A**) and amlodipine-treated group (**C**)

**Fig. 4:**
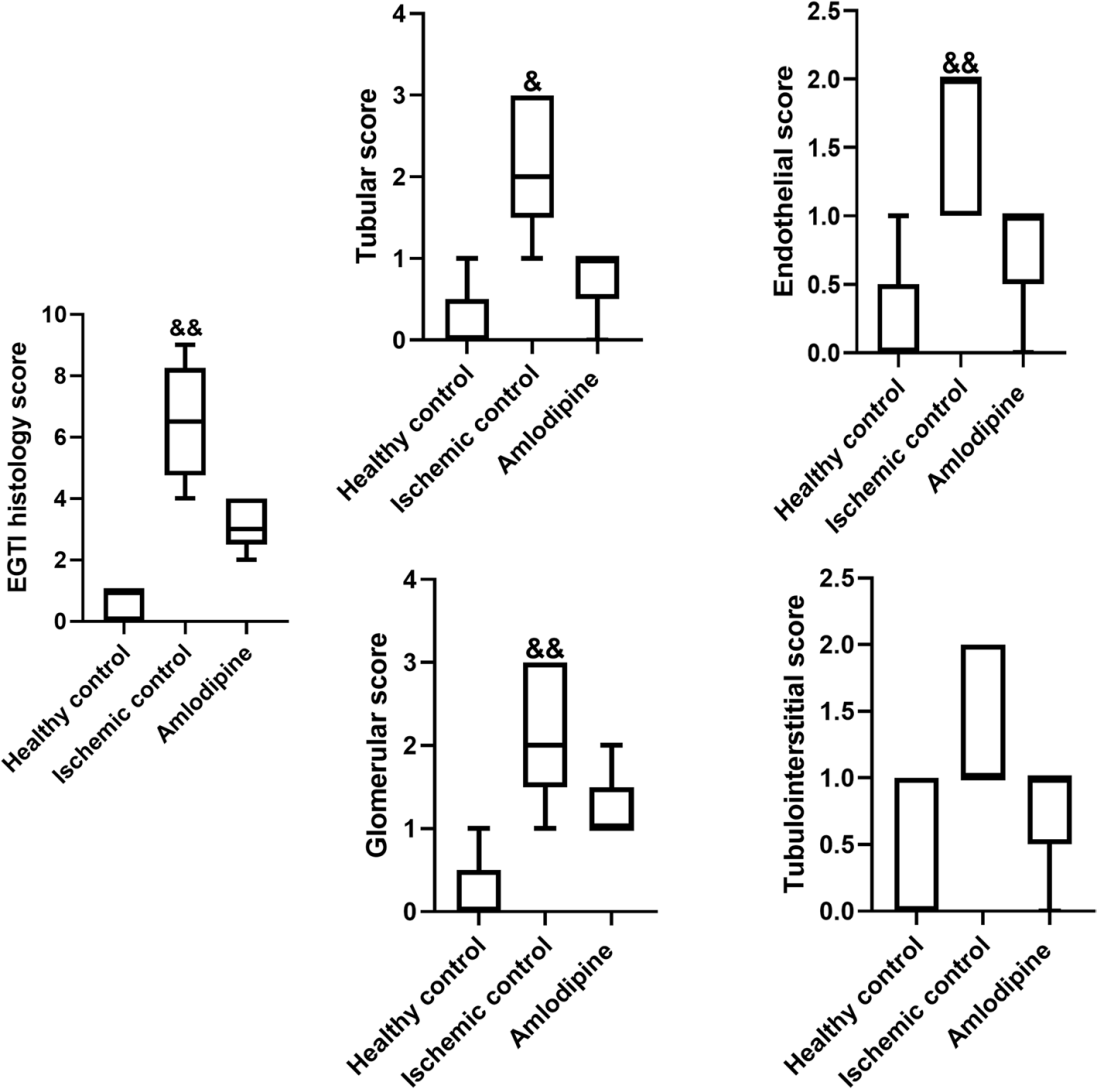
EGTI histology score of specimens after renal I/R injury. Renal I/R injury was associated with a marked increase in EGTI histology score (&&, *p* < 0.01), but amlodipine did not significantly decrease the histology score. I/R injury significantly increased tubular (&, *p* < 0.05), endothelial (&&, *p* < 0.01), and glomerular (&&, *p* < 0.01) scores. Treatment with amlodipine did not lower tubular, endothelial, glomerular, and tubulointerstitial scores. *Kruskal–Wallis* test and Dunn’s posthoc test were used to compare histological scores. Box and Whisker plots show median, 25 and 75 percentiles, minimum, and maximum values. Median (interquartile range) for EGTI histology score were 0 (0, 1), 7 (5.5, 8.5), and 3 (2.5, 4) in the healthy control group, ischemic control group, and amlodipine-treated group, respectively. Median (interquartile range) for tubular score were 0 (0, 0.5), 2 (1.5, 3), and 1 (0.5, 1) in the healthy control group, ischemic control group, and amlodipine-treated group, respectively. Median (interquartile range) for glomerular score were 0 (0, 0.5), 2 (1.5, 3), and 1 (1, 1.5) in the healthy control group, ischemic control group, and amlodipine-treated group, respectively. Median (interquartile range) for endothelial score were 0 (0, 0.5), 2 (1, 2), and 1 (0.5, 1) in the healthy control group, ischemic control group, and amlodipine-treated group, respectively. Median (interquartile range) for tubulointerstitial score were 0 (0, 1), 1 (1, 2), and 1 (0.5, 1) in the healthy control group, ischemic control group, and amlodipine-treated group, respectively

After tissue processing, specimens were stained with H&E. Each sample received a score, based on the endothelial, glomerular, tubular, and interstitial (EGTI) histology scoring system. Signs of tissue damage such as inflammation, hemorrhage, necrosis of tubular cells and thickening of Bowman capsule, retraction of glomerular tuft, and glomerular fibrosis were observed in the ischemic control group and partially recovered in the amlodipine-treated group (Fig. 3). Renal I/R injury markedly increased the EGTI histology score (&&, *p* < 0.01), but amlodipine could not significantly decrease the histology score. I/R injury significantly increased tubular (&, *p* < 0.05), endothelial (&&, *p* < 0.01), and glomerular (&&, *p* < 0.01) scores. Treatment with amlodipine did not lower tubular, endothelial, glomerular, and tubulointerstitial scores (Fig. 4).

### Amlodipine significantly decreased inflammatory cytokines and MDA levels

**Fig. 5:**
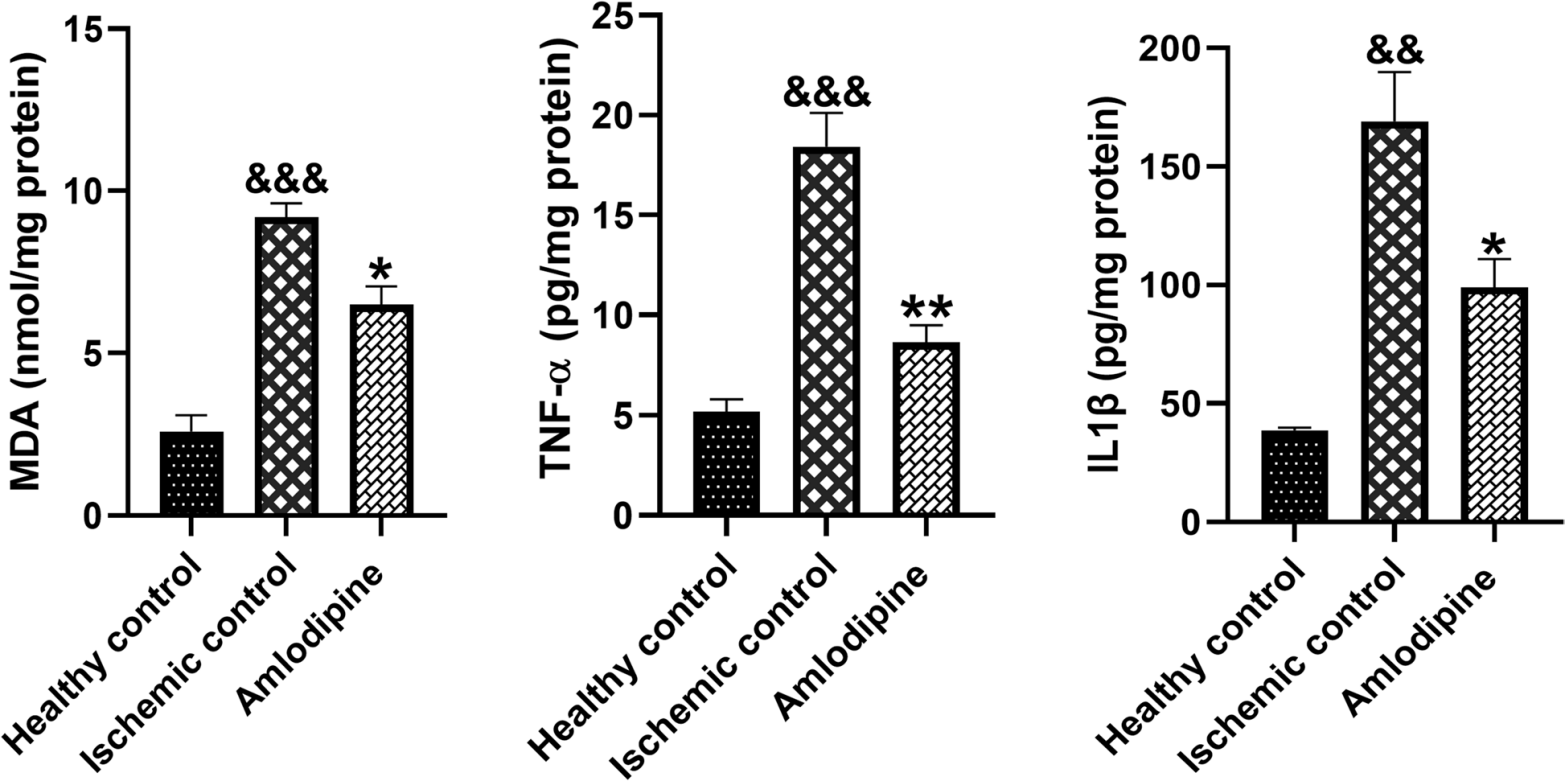
The effect of amlodipine on lipid peroxidation and inflammatory markers in renal I/R injury. I/R injury led to a significant increase in MDA (&&&, *p* < 0.001), TNF-α (&&&, *p* < 0.001) and IL1β (&&, *p* < 0.01) levels in tissue specimens. MDA (*, < 0.05), TNF-α (**, *p* < 0.01), and IL1β (*, *p* < 0.05) levels remarkably decreased after treatment with amlodipine. One-way analysis of variance (ANOVAs) followed by post hoc Tukey’s tests was used to compare the groups. Charts are shown as mean ± standard error of the mean (SEM) (*n* = 5)

The tissue levels of MDA, IL1β, and TNF-α were measured in tissue samples. As the marker of oxidative stress, MDA level was significantly (&&&, *p* < 0.001) increased in the ischemic control group. In addition, I/R injury increased the tissue levels of IL1β (&&, *p* < 0.01) and TNF-α (&&&, *p* < 0.001). Treatment with amlodipine significantly decreased the tissue levels of MDA (*, < 0.05), TNF-α (**, *p* < 0.01), and IL1β (*, *p* < 0.05) in the kidney biopsies (Fig. 5).

### Amlodipine upregulated the main regulators of antioxidant defense in I/R injury

**Fig. 6:**
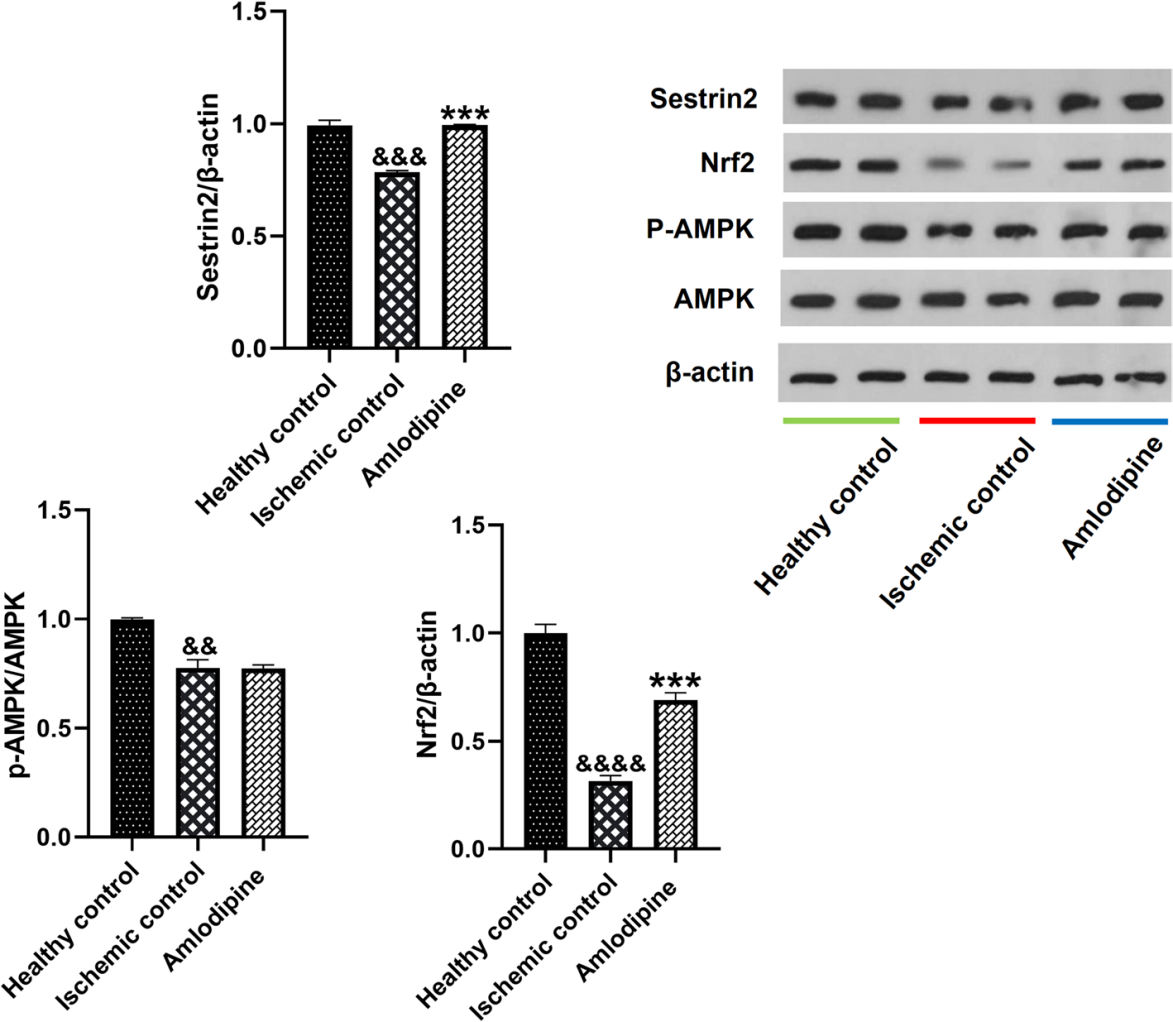
The effect of amlodipine on the expression of master regulators of antioxidant response and metabolism. Renal I/R injury has been associated with a significant decrease in Sestrin2/β-actin (&&&, *p* < 0.001), Nrf2/β-actin (&&&&, *p* < 0.0001), and p-AMPK/AMPK (&&, *P* < 0.01). Treatment with amlodipine enhanced the expression of Nrf2 (***, *p* < 0.001) and Sestrin2 (***, *p* < 0.001), whereas did not change AMPK phosphorylation. One-way analysis of variance (ANOVAs) followed by post hoc Tukey’s tests was used to compare the groups. Charts are shown as mean ± standard error of the mean (SEM) (*n* = 3)

Tissue biopsies were snap-frozen and kept at -80 °C until assay. Western blotting was used to measure p-AMPK/AMPK, Sestrin2/β-actin, and Nrf2/β-actin ratios. I/R injury notably downregulated Sestrin2/β-actin (&&&, *p* < 0.001), Nrf2/β-actin (&&&&, *p* < 0.0001) and p-AMPK/AMPK (&&, *P* < 0.01). Treatment with amlodipine could not make a significant change in the p-AMPK/AMPK ratio, whereas led to a significant increase in the expression of Sestrin2 (***, *p* < 0.001) and Nrf2 (***, *p* < 0.001) (Fig. 6 and Supplementary Material 1).

### Amlodipine augmented autophagy, improved mitochondrial biogenesis, and prevented apoptosis in renal I/R injury

**Fig. 7:**
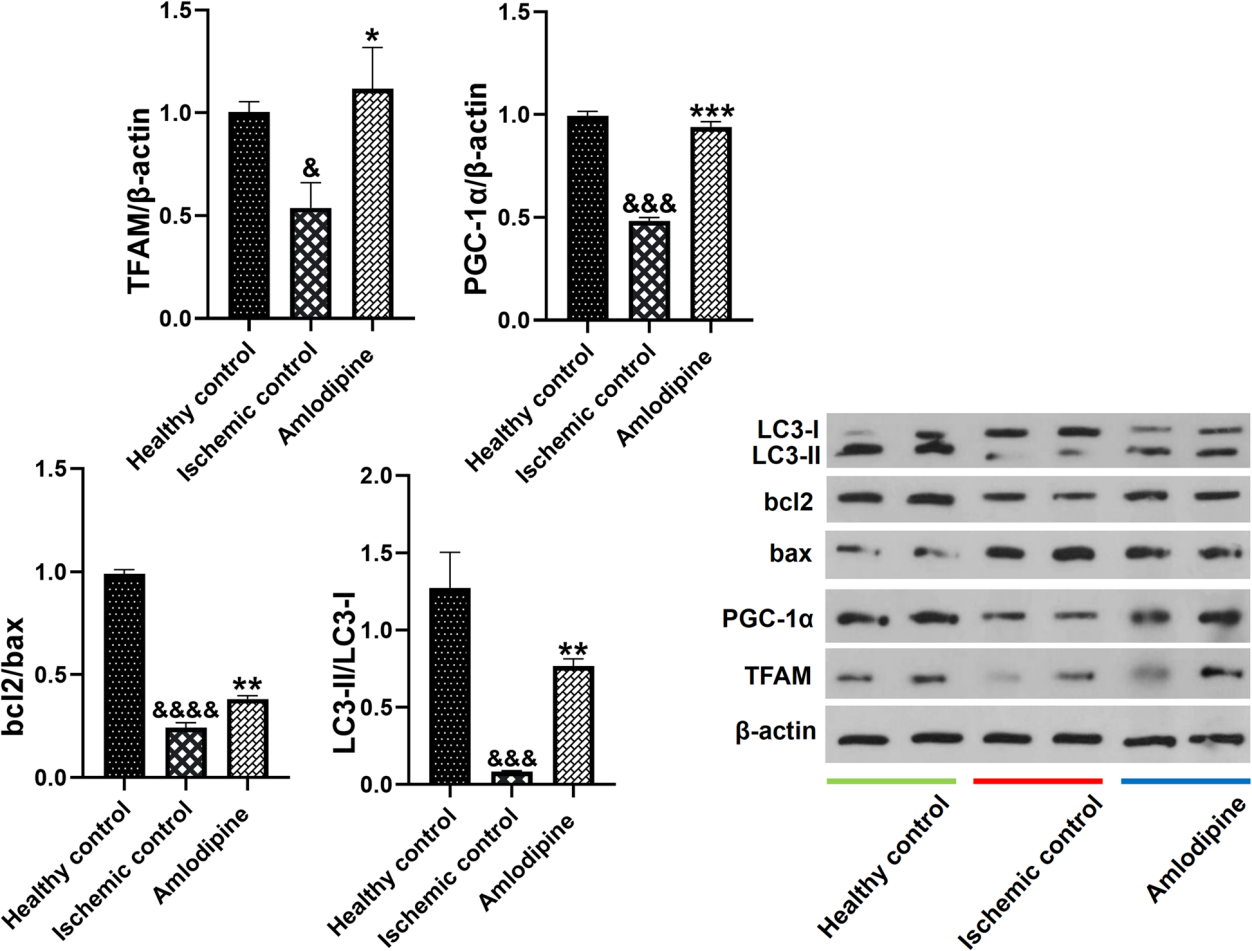
The effect of I/R injury and amlodipine treatment on autophagy, mitochondrial biogenesis, and apoptosis in the kidney. I/R injury decreased bcl2/bax (&&&&, *p* < 0.0001), LC3-II/LC3-I (&&&, *p* < 0.001), TFAM/β-actin (&, *p* < 0.05), and PGC-1α/β-actin (&&&, *p* < 0.001). In contrast, treatment with amlodipine significantly increased bcl2/bax (**, *p* < 0.01), LC3-II/LC3-I (**, *p* < 0.01), TFAM/β-actin (*, *p* < 0.05), and PGC-1α/β-actin (***, *p* < 0.001). One-way analysis of variance (ANOVAs) followed by post hoc Tukey’s tests was used to compare the groups. Charts are shown as mean ± standard error of the mean (SEM) (*n* = 3)

Western blotting was also used to measure the markers of apoptosis, autophagy, and mitochondrial biogenesis. Herein, the expression levels of bcl2, bax, LC3-I, LC3-II, PGC-1α, and mitochondrial transcription factor A (TFAM) were measured. I/R injury led to a pronounced decrease in bcl2/bax (&&&&, *p* < 0.0001), LC3-II/LC3-I (&&&, *p* < 0.001), TFAM/β-actin (&, *p* < 0.05), and PGC-1α/β-actin (&&&, *p* < 0.001), showing that renal I/R has been associated with increased apoptosis and impaired autophagy and mitochondrial biogenesis. Treatment with amlodipine vigorously reversed these alterations and increased bcl2/bax (**, *p* < 0.01), LC3-II/LC3-I (**, *p* < 0.01), TFAM/β-actin (*, *p* < 0.05), and PGC-1α/β-actin (***, *p* < 0.001) (Fig. 7 and Supplementary Material 1).

## Discussion

The current study showed that amlodipine improves renal I/R injury in rats, preserves renal function, maintains tissue microarchitecture, and prevents glomerular and tubulointerstitial damage. Amlodipine vigorously enhanced autophagy and mitochondrial biogenesis and suppressed oxidative stress, inflammatory cytokines release, and apoptosis.

Halici et al*.* revealed that amlodipine can mitigate ovarian I/R injury in rats [15]. They observed that amlodipine can attenuate oxidative stress in ovarian I/R injury [15]. Likewise, amlodipine alleviated myocardial I/R injury in pigs by mitigating oxidative stress and calcium overload [21]. Uncontrolled production of ROS subsequent to impaired mitochondrial function is a major propellant of organ damage during I/R injury [22, 23]. Except for their direct damage to cell structure, ROS activate several inflammatory pathways and lead to apoptosis [22, 23]. Oxygen depletion during the ischemic phase decreases adenosine triphosphate (ATP) synthesis [24, 25]. In the absence of enough amount of ATP, Na^+^/K^+^ ATPase cannot decrease intracellular Na^+^ concentration [25]. Subsequently, Na^+^/Ca^2+^ anti-porter cannot effectively exchange extracellular Na^+^ with intracellular Ca^2+^; therefore, Ca^2+^ accumulates in the intracellular space [24, 25]. Likewise, redistribution of endoplasmic reticulum Ca^2+^ increases the cytosolic concentration of Ca^2+^ [24, 25]. Intracellular deposition of Ca^2+^ results in the activation of a wide variety of destructive enzymes such as protease, phospholipase A2, and endonuclease which ignites apoptosis mechanisms [24]. Ca^2+^ influx also stimulates ROS production in inflammation [26]. In a Ca^2+^-overloaded cell with impaired mitochondrial function, replenishment of depleted oxygen supply aggravates ROS production in the reperfusion phase [24, 25]. Based on this theory, Ca^2+^ channel blockade can be a good strategy to prevent cellular damage during renal I/R injury, as amlodipine did in this study.

Similar to this study, previous studies have shown that amlodipine can decrease MDA levels [16, 27]. Newly, this study has shown that amlodipine can strongly upregulate Nrf2 and Sestrin2 which are the main regulators of antioxidant defense [28]. Nrf2 is a transcription factor for numerous endogenous antioxidants such as Sestrin2. Nrf2 hyperactivation was shown to protect against tubular damage in renal I/R [28, 29]. Sestrin2 is a downstream molecule of Nrf2, on the other hand, it is needed for autophagic degradation of Kelch-like ECH-associated protein 1 (Keap1) and for releasing Nrf2 [30, 31]. By releasing Nrf2 from Keap1 sequestration, Sestrin2 plays a crucial role in antioxidant defense [31]. Except for increasing ROS scavenging, upregulation of Sestrin2 can alleviate renal I/R injury by reinforcing autophagy and mitophagy to mitigate mitochondrial dysfunction [32–34].

Sestrin2 can improve mitochondrial biogenesis [35]. Ineffective mitochondrial biogenesis is deeply involved in the pathogenesis of AKI and abnormal kidney repair [36]. Sestrin2 upregulates PGC-1α in I/R injury to improve mitochondrial biogenesis [33, 37]. PGC-1α is a transcriptional factor and a chief regulator of mitochondrial biogenesis [38, 39]. Sufficient expression of PGC-1α can protect renal tubular cells against energy deficit, AKI, and CKD [38]. Similar to PGC-1α, AMPK is involved in mitochondrial biogenesis [33, 35], but amlodipine could not significantly affect AMPK expression or activation in this study.

TFAM is downstream of PGC-1α, preserves mitochondrial DNA against harmful stimuli, and protects against renal I/R injury [40]. Mitochondrial ROS decrease TFAM expression, expedites its degradation, and endangers the integrity of mitochondrial DNA [40]. In exchange, increased expression and stability of TFAM can maintain mitochondrial DNA integrity and reduce inflammation and ROS production in I/R injury [41]. Consistently, decreased renal expression of TFAM is a common finding in patients who experience AKI [40]. In this study, amlodipine markedly promoted the expression of PGC-1α and TFAM to modulate mitochondrial biogenesis and function during renal I/R injury.

Based on these findings amlodipine can confine oxidative stress in renal I/R injury through two mechanisms: 1- Improving mitochondrial function and decreasing ROS production. 2- Empowering antioxidant defense.

Amlodipine markedly enhanced the LC3-II/LC3-I ratio in this study which is an indicator of autophagy [42]. It was shown that augmentation of autophagy can decrease inflammation and tissue damage and reduce the tissue levels of TNF-α and IL6 in renal I/R injury [43]. Consistently, it was shown that inhibition of autophagy exacerbates the outcome of renal I/R injury [43, 44]. Previously, it has been shown that upregulation of Sestrin2 can activate autophagy in AKI [45]. Autophagy is an opportunity for renal tubular cells to restore themselves, remove dysfunctional organelles and misfolded proteins, and prevent apoptotic cell death [44].

These findings suggest that amlodipine can effectively ameliorate renal I/R injury. Augmentation of autophagy and mitochondrial biogenesis and attenuation of oxidative stress, inflammation, and apoptosis were found to be involved in the protective effect of amlodipine on renal I/R injury. Regarding the promising effects of amlodipine on contrast- and drug-induced AKI in human studies and its beneficial effects in animal studies of renal I/R injury, it is worth measuring the effect of amlodipine in clinical trials of AKI.

### Supplementary Information

**Additional file 1: Supplementary Material 1.** Uncropped original blotting images provided in this article.

## Data Availability

Data analyzed for this article will be available from the corresponding author by a reasonable request.
