# Supplementary material for: Amlodipine alleviates renal ischemia/reperfusion injury in rats through Nrf2/Sestrin2/PGC-1α/TFAM Pathway
Source: BMC Pharmacol Toxicol. 2023 Dec 21;24:82. doi: 10.1186/s40360-023-00722-6 (PMC10740300; doi:10.1186/s40360-023-00722-6)

Supplementary File 1. Western blotting gels were not cropped from several points or taken from different gels for showing in the manuscript. The gels viewed in the article were located adjacent to each other in the original gel, as shown in the figures. All figures follow the same sequence of samples.

B-actin

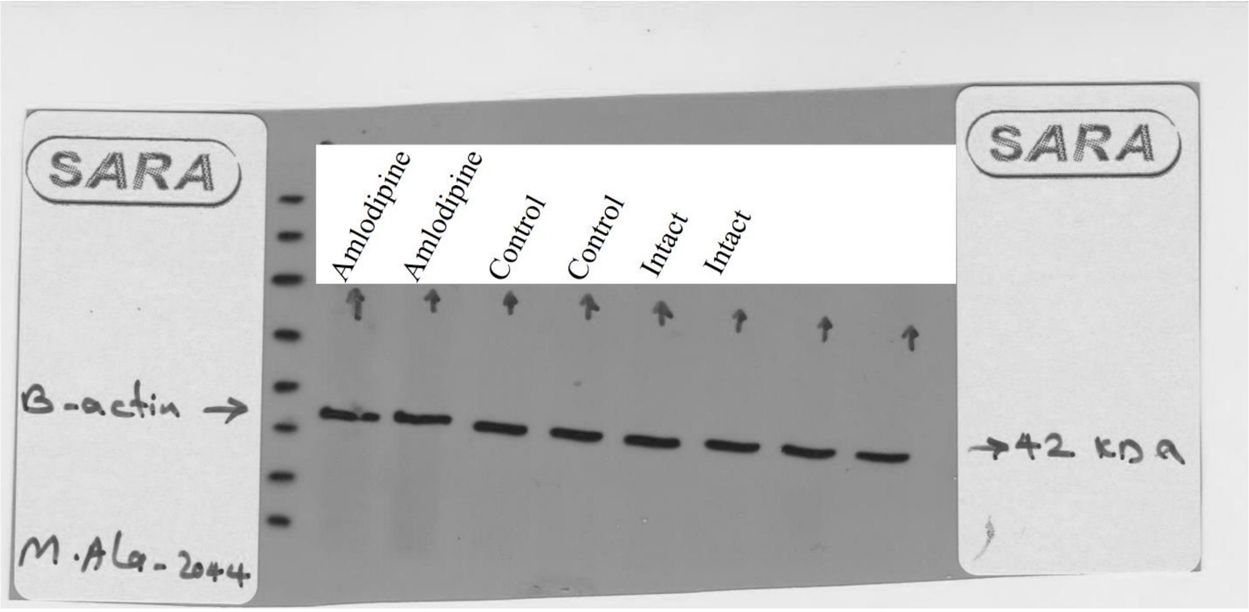

AMPK

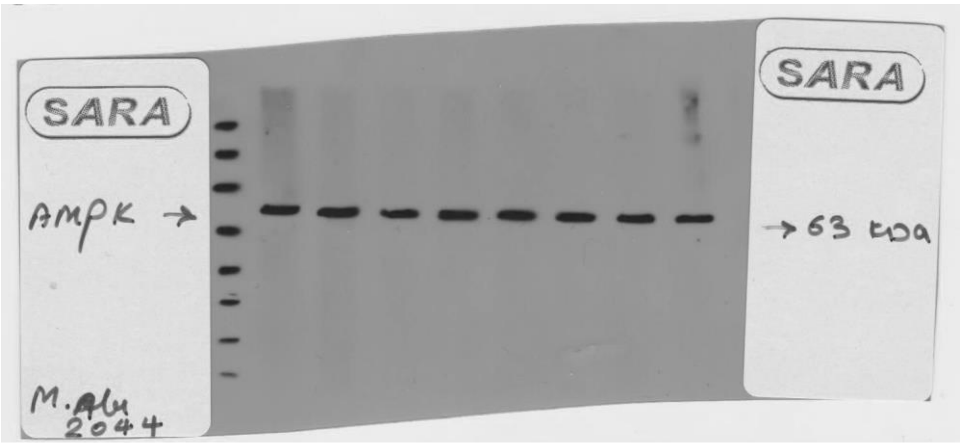
SARA
SARA
AMPK →
→ 63 kDa
M. Ala
2044

p-AMPK

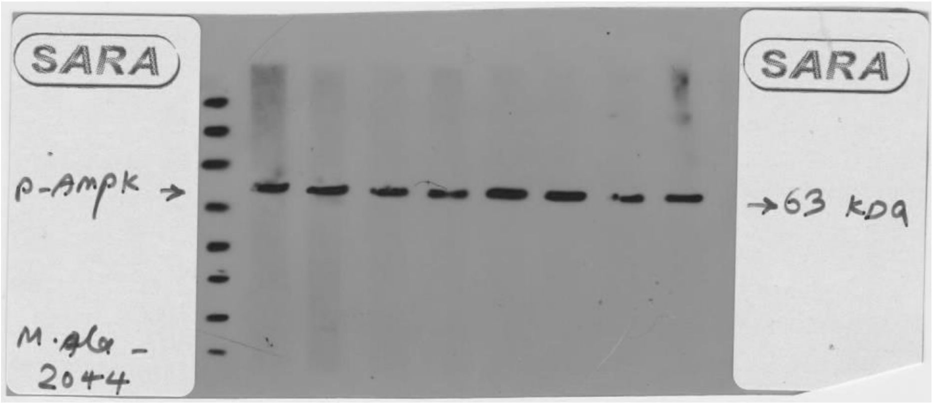
SARA
SARA
p-AMPK →
→ 63 kDa
M. Ala -
2044

Bax

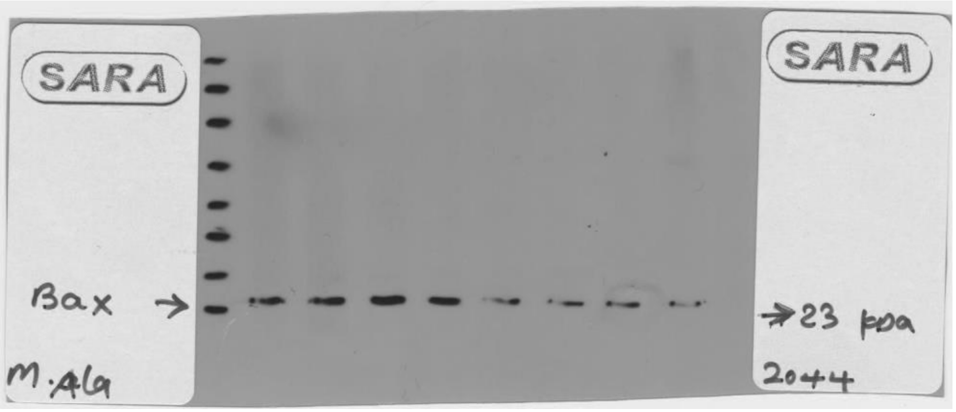

Bcl2

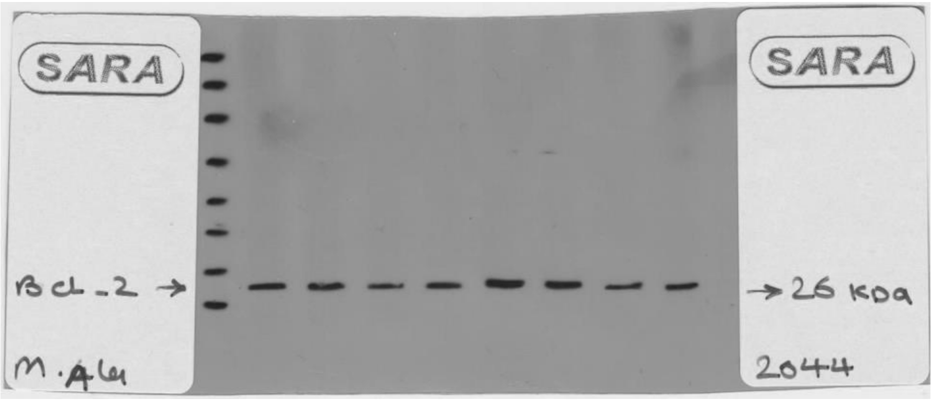

LC3I, II

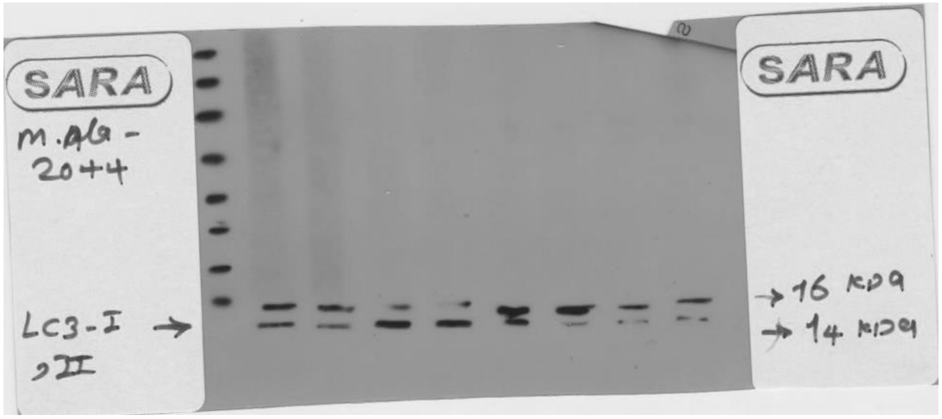
SARA
m.Ala-
2044
LC3-I
,II
SARA
16 kDa
14 kDa

Nrf2

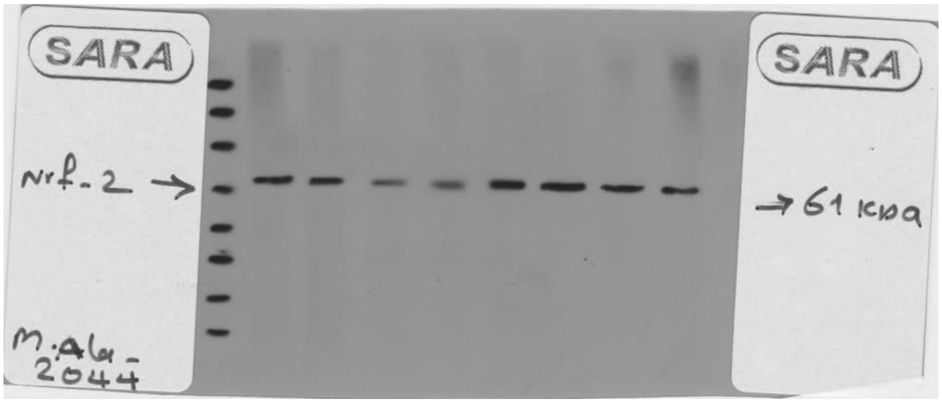
SARA
Nrf-2
m.Ala-
2044
SARA
61 kDa

PGC-1α

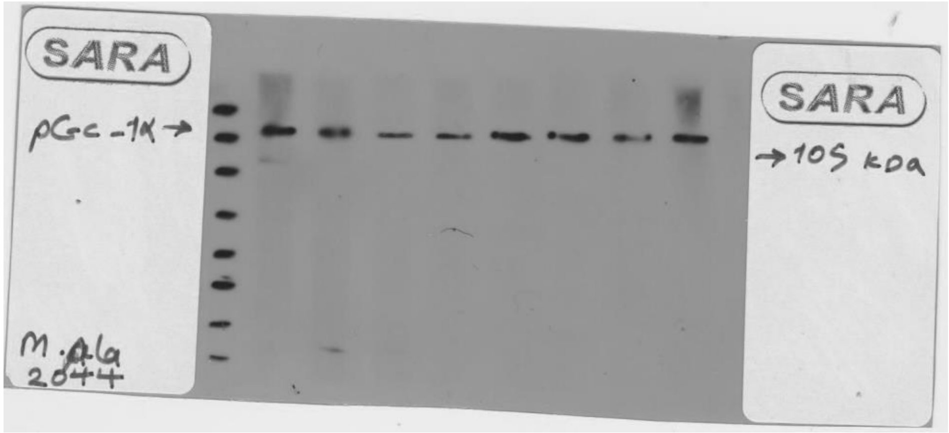

Sestrin2

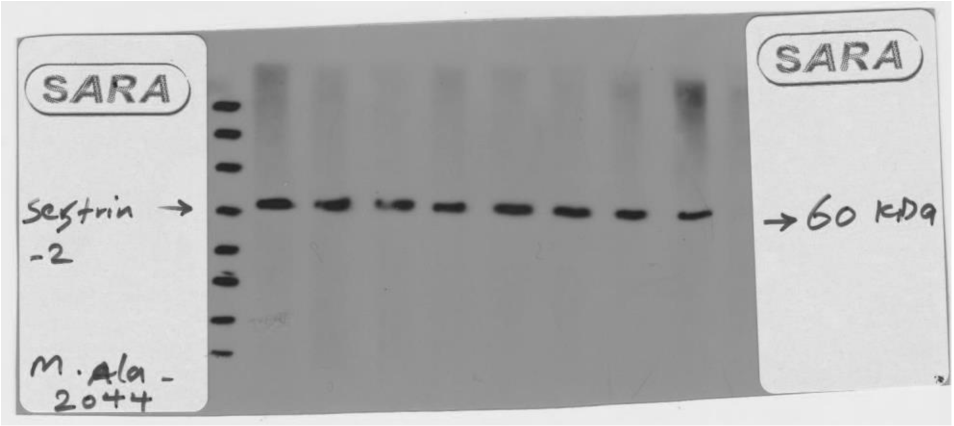

TFAM

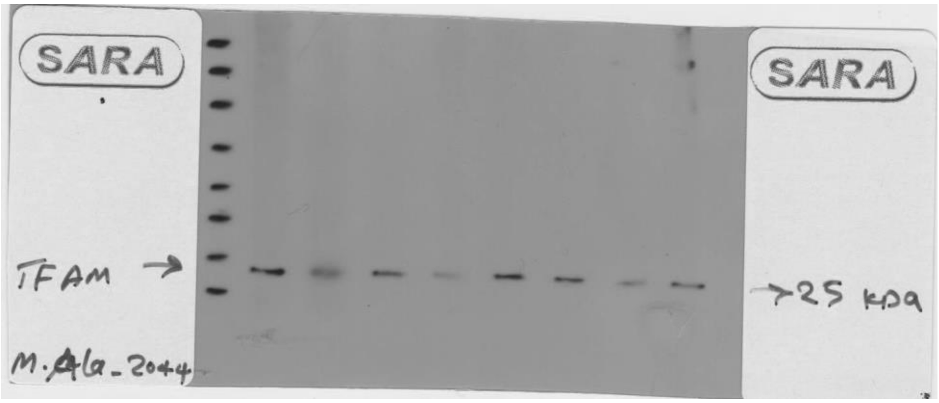

Supplement: Supplementary file 1 — Additional file 1: Supplementary Material 1. Uncropped original blotting images provided in this article. [file 40360_2023_722_MOESM1_ESM.pdf]
